# New horizons in Biophysics

**DOI:** 10.1186/2046-1682-4-1

**Published:** 2011-03-02

**Authors:** Elizabeth C Moylan

**Affiliations:** 1 Biology Editor, BMC series journals

## Abstract

This editorial celebrates the re-launch of *PMC Biophysics *previously published by PhysMath Central, in its new format as *BMC Biophysics *published by BioMed Central with an expanded scope and Editorial Board. *BMC Biophysics *will fill its own niche in the *BMC *series alongside complementary companion journals including *BMC Bioinformatics, BMC Medical Physics*, *BMC Structural Biology *and *BMC Systems Biology*.

## Editorial

This month *PMC Biophysics *joins the *BMC *series of journals as *BMC Biophysics*. The 'P to B' transition is not some strange quirk of molecular dynamics but the consequence of integrating PhysMath Central journals into Springer and BioMed Central portfolios which has resulted in a very welcome addition to the *BMC *series. Previously, *PMC Biophysics *had been published by PhysMath Central but will now be published by BioMed Central - as is the case with all journals in the *BMC *series. When BioMed Central was founded in 2000, the *BMC *series of journals were among the first to be launched. Since then, this portfolio of journals have grown rapidly and become well-recognised in the research communities they serve. We are committed to the future of *BMC Biophysics *and the journal will be in good company among the many successful titles in the *BMC *series.

Under the stewardship of its Editor-in-Chief, Huan-Xiang Zhou, *PMC Biophysics *was originally launched in 2008 in response to the gathering strength of the open access movement and the multidisciplinary nature of research in biological physics [1]. *BMC Biophysics *will maintain this ethos, and continue to publish articles in experimental and theoretical aspects of biological processes from the microscopic to macroscopic level. Topics include (but are not limited to) thermodynamics, structural stability and dynamics of biological macromolecules and mesoscale cellular processes. We also welcome studies on membrane biophysics, nucleic acids, signalling and interaction networks and novel biophysical methods. In keeping with recent developments, we have also broadened the scope of the journal to explicitly cover computational and theoretical biophysics.

As with the other journals in the *BMC*-series stable, *BMC Biophysics *has an international Editorial Board which retains much of the *PMC Biophysics *Editorial Board with additional new faces [2] and comprises Section Editors, Associate Editors and Editorial Advisors. We are delighted that Huan-Xiang Zhou will continue his strong involvement with the journal as a Section Editor and he provides his personal perspective on biophysics in a forthcoming 'Question and Answer' piece in *BMC Biology *[3].

All previous *PMC Biophysics *content will remain open access and will be freely accessible from the new *BMC Biophysics *platform. New submissions to the journal will be assessed by our Section Editors for their suitability for peer review and our Section Editors will work closely with our Associate Editors to facilitate the review process and ensure consistency of decision making. The journal is further supported by a group of Editorial Advisors with specialist knowledge in particular fields. Together the Editorial Board will liaise with in-house Editorial staff to maintain journal thresholds and policies, and to steer the future of *BMC Biophysics*.

Despite recent government funding cuts, which can often hit interdisciplinary research areas the hardest, biophysics is rapidly gaining momentum. There is of course a history of physics in biology but the need for collaboration is still strong today, from such diverse fields as mapping cancer cell behaviour [4] to novel ideas for antenna structures and nanoparticle synthesis in the field of single molecule biophysics [5]. It's also heartening to see that publishers have recognised the need to cater for research in the physical sciences by providing open access venues. The recent launch of SpringerOpen [6] provides a range of fully open access journals which gives authors in Physics, Chemistry, Mathematics, Computer Science, and Social Sciences the opportunity to publish open access articles. The announcement by the American Physical Society of their forthcoming journal Physical Review X [7] also shows commitment to the recent research growth in physics and its application to related fields.

We are of course preaching to the converted when highlighting the benefits of publishing open access to the physics community who have long accepted the advantages offered by self-archiving in arXiv.org [8]. However, although arXiv.org provides a means of circulating manuscripts, it does not offer the benefits and visibility to the biological community that publishing a peer reviewed article with *BMC Biophysics *can provide. Articles published in *BMC Biophysics *will of course be open access and be freely and universally accessible online immediately upon acceptance, ensuring that research is disseminated to the widest possible audience. In order to cover the costs of publication, an article-processing charge (APC) is levied to authors or their institution on acceptance of the article. This flat fee is among the lowest charged across other publishers offering open access [9]. Publishing in electronic format allows the full use of digital technologies and permits the inclusion of large data sets, links to other web pages, animations, slide shows, video clips and unlimited colour, all at no additional charge. If the institute of the author submitting a manuscript is a member of BioMed Central the cost of the APC is covered by the membership and no further charge is payable [10]. We are able to grant waivers under special circumstances and a number of funding agencies allow their grants to be used for APCs [11]. Finally, *BMC Biophysics *also welcomes initiatives that encourage standardized reporting and public deposition of datasets [12,13].

**Figure 1:**
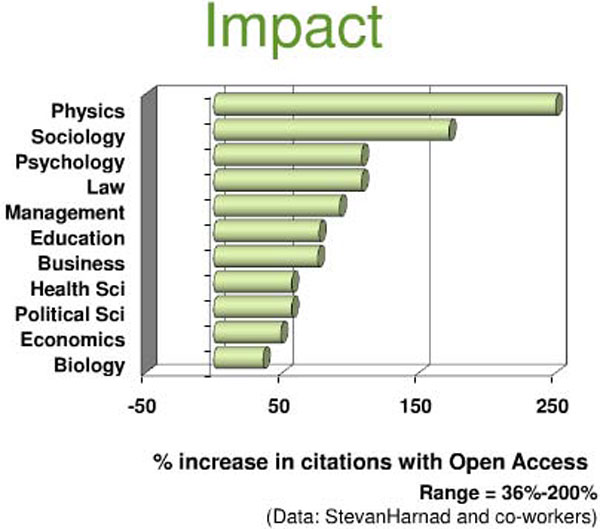
**The impact of publishing open access is apparent from the % increase in citations from open access articles**. This is greatest in the field of physics and least in biology. Figure taken from [17] which was presented under a Creative Commons Attribution-Share Alike 3.0 Unported License.

Research published open access has the potential to reach a much wider range of readers than any subscription-based journal, either in print or online [14]. And of course if readers can freely access articles this leads to more downloads and more citations, leading to a higher Impact Factor [15,16]. As re-emphasised recently by Alma Swan [17] when discussing a study by Steven Harnad and Tim Brody [18] in which they compared the citation counts of individual open access and non-open access articles appearing in the same (non-open access) journals: there is a clear citation advantage to publishing open access. This open access citation effect is strongest in physics and least in biology (Figure 1). Although it's not obvious where 'biophysicists' sit in the spectrum, open access publishing is clearly the best way for authors to maximise the impact of their work.

We are delighted to be relaunching *BMC Biophysics *with an inaugural thematic series on 'Biological diffusion and Brownian dynamics' guest edited by Rebecca Wade, Paolo Mereghetti and Andy McCammon. We also take this opportunity to encourage researchers to consider the journal for publishing their own results of workshop meetings. In these fast-moving times, where researchers from many fields are coming together to make up the interdisciplinary field of Biophysics, there is a clear need for the scientific community to keep abreast of the latest developments in an open-access format. So, if you are a physicist working on biological systems, or a biologist using the tools of physics, or a biochemist somewhere on the interface of these disciplines we do hope you will take the time to read more about the journal and consider us for your future submissions.
